# Slope-Reducing Tibial Plateau-Leveling Osteotomy

**DOI:** 10.1016/j.eats.2024.103264

**Published:** 2024-09-22

**Authors:** Matthieu Ollivier, Alexander J. Hoffer, Shintaro Onishi, Jean Brilhault, Solenne Frey-Ollivier, Brian Godshaw, Kristian Kley

**Affiliations:** a Institute of Movement Sciences, Sainte-Marguerite Hospital, Aix-Marseille University, Marseille, France; b Department of Orthopedic Surgery, Mayo Clinic Arizona, Phoenix, Arizona, U.S.A.; c Department of Orthopaedic Surgery, Hyogo College of Medicine, Hyōgo, Japan; d Centre Cheville & Pied, Clinique St Léonard, Trélazé, France; e Centre du Pied, Marseille, France; f Ochsner Andrews Sports Medicine Institute, New Orleans, Louisiana, U.S.A.; g Orthopaedic Care Center, Harley Street Specialist Hospital, London, England

## Abstract

Increased posterior tibial slope (PTS) is a risk factor for anterior cruciate ligament (ACL) tear and ACL reconstruction failure. Anterior closing-wedge osteotomy (ACWO) decreases the PTS and the risk of reinjury after revision ACL reconstruction. ACWO techniques include supratubercular, transtubercular, or infratubercular. However, there are limitations to the amount of slope correction an ACWO can achieve in the context of a massively abnormal slope. If the necessary slope correction is greater than 20°, a supratubercular ACWO cannot achieve the required correction without high risk of brittle fixation, a transtubercular ACWO would introduce increased morbidity of a secondary osteotomy and change the biomechanics of the patellofemoral joint resulting in significant patella alta, and an infratubercular ACWO would require a major anterior tibial metaphysodiaphyseal resection. The tibial plateau leveling osteotomy (TPLO) is an alternative curved osteotomy commonly used in canines that avoids the adverse events associated with a large ACWO. Further benefits of TPLO for massive slope correction include improved maintenance of native soft-tissue tension, avoidance of major recurvatum, and the ability to control the correct degree. We describe an open technique for a TPLO to decrease the PTS and the risk of recurrent ACL injury.

Anterior cruciate ligament reconstruction (ACLR) is one of the most frequently performed orthopaedic surgical procedures, with an estimated 350,000 undertaken annually in the United States.^1^ However, the risk of reinjury after ACLR is up to 18% in a high-risk population.^2^ An abnormal sagittal proximal tibial slope has been identified as a risk factor for ACLR failure.^3^^,^^4^ Reduction of the sagittal slope by anterior closing-wedge osteotomy (ACWO) of the proximal tibia may be an effective technique to decrease the risk of ACLR failure.^5^

ACWO of the proximal tibia may be performed above the tibial tubercle (supratubercular), at the level of the tibial tubercle (transtubercular) with a concomitant tibial tubercle osteotomy, or below the level of the tibial tubercle (infratubercular).^6^ Each technique has its own benefits and drawbacks.^7^ However, none of the current techniques can appropriately achieve a large slope correction (>20°) without a significant risk of complications. A supratubercular ACWO cannot achieve such a large correction without brittle fixation and resultant patella alta.^8^ A transtubercular osteotomy results in metaphyseal recurvatum, patella alta, and added morbidity of a secondary osteotomy. An infratubercular osteotomy results in metaphysodiaphyseal recurvatum with major tibial bone resection, putting the tibia at risk for secondary fracture. A tibial plateau-leveling osteotomy (TPLO) is an alternative strategy to correct a massively abnormal sagittal proximal tibial slope. The TPLO is a curved, reverse-dome osteotomy and is a common treatment option in canines with cranial cruciate ligament tears to correct their naturally large sagittal slope.^9^, ^10^, ^11^ We describe a technique for TPLO to correct a massively abnormal sagittal slope using orthopaedic instrumentation that is readily available in a common orthopaedic center. All procedures were performed in compliance with relevant laws and institutional guidelines. No institutional review board approval was needed for this nonexperimental surgical innovation. Informed consent was obtained for this nonexperimental surgical innovation.

## Surgical Technique

A detailed video of the TPLO technique is shown in Video 1. Surgical pearls and pitfalls of this procedure are discussed in Table 1.

**Table 1:** Pearls and Pitfalls of Slope-Reducing Tibial Plateau-Leveling Osteotomy

Pearls	Pitfalls
Preoperative planning is essential to achieve the desired correction without introducing a secondary deformity.	Failure to identify the correct osteotomy curvature preoperatively may result in the introduction of angular deformity or significant osteotomy step-off.
Subperiosteal dissection of the anterior tibial compartment ensures no unwanted neurovascular injury during access to the proximal tibial-fibular joint.	Intramuscular dissection in the anterior tibial compartment may result in unwanted bleeding.
Obtain a perfect lateral fluoroscopic view of the proximal tibia to identify the center of the metaphysis and the rotation point.	Failure to use fluoroscopy may result in a suboptimal osteotomy and resultant correction.
Use dual-plate fixation to maximize the construct strength, allow for early motion, and to minimize knee stiffness.	The use of one plate or staples alone may compromise the construct strength and result in unwanted complications if the patient bears weight early.

### Step 1: Preoperative Workup

The workup for instability associated with anterior cruciate ligament (ACL) deficiency includes a detailed history, physical examination, and radiographs. A dedicated knee series including anterior-posterior, lateral, Rosenberg, and hips-to-ankles alignment radiographs are mandatory. Magnetic resonance imaging is indicated to diagnose associated soft-tissue abnormalities. If nonoperative management options including physical therapy, oral analgesics, and injections are exhausted, then surgical management is indicated.

### Step 2: Operative Planning

**Fig 1:**
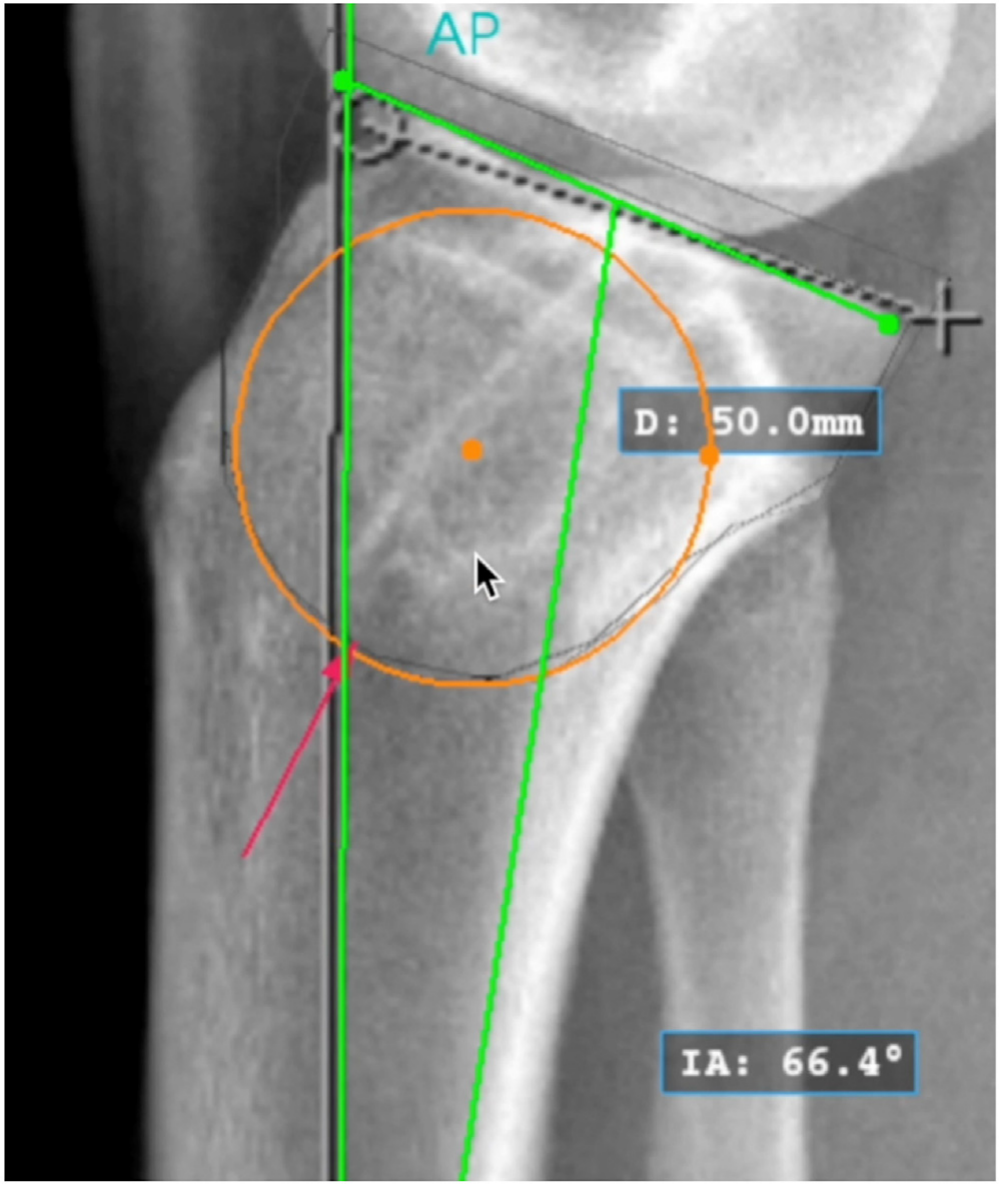
A lateral radiograph of the right knee is used to identify the center of the proximal tibia (orange dot) using a best-fit circle (orange circle). The sagittal slope is measured using the anatomic axis (vertical green line with red arrow) and medial tibial plateau (green line) (IA: 66.4°). (D: 50.0 mm) is the diameter of the best-fit circle. (D, diameter; IA, posterior tibial slope measure.)

**Fig 2:**
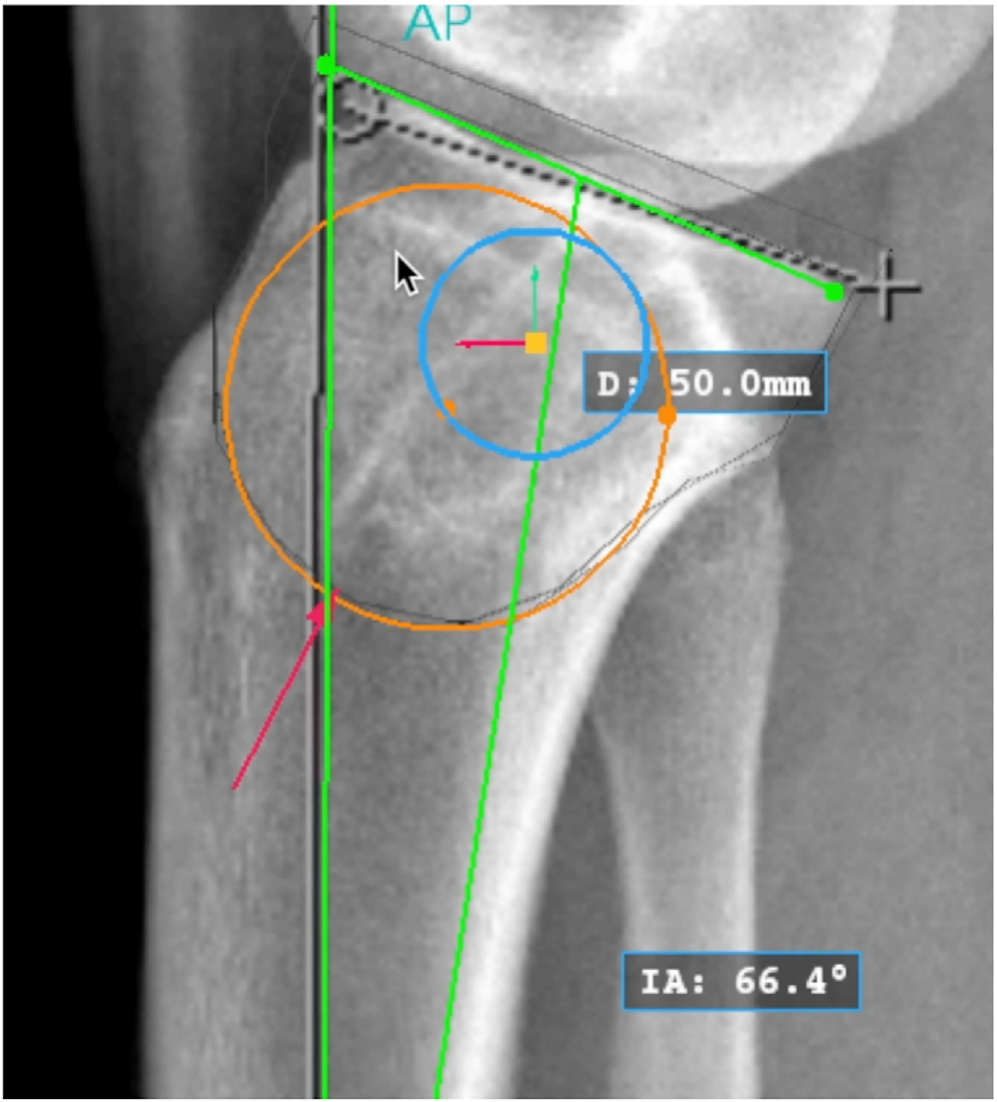
A lateral radiograph of the right knee shows the osteotomy center of rotation just above the proximal tibial-fibular joint, identified by the yellow dot. The osteotomy radius of rotation is identified by the blue circle, which subtends the center of the proximal tibia (orange dot at the center of the orange circle). The sagittal slope is measured using the anatomic axis (vertical green line with red arrow) and medial tibial plateau (IA: 66.4°). (D: 50.0mm) is the diameter of the best-fit circle. (D, diameter; IA, posterior tibial slope measure.)

**Fig 3:**
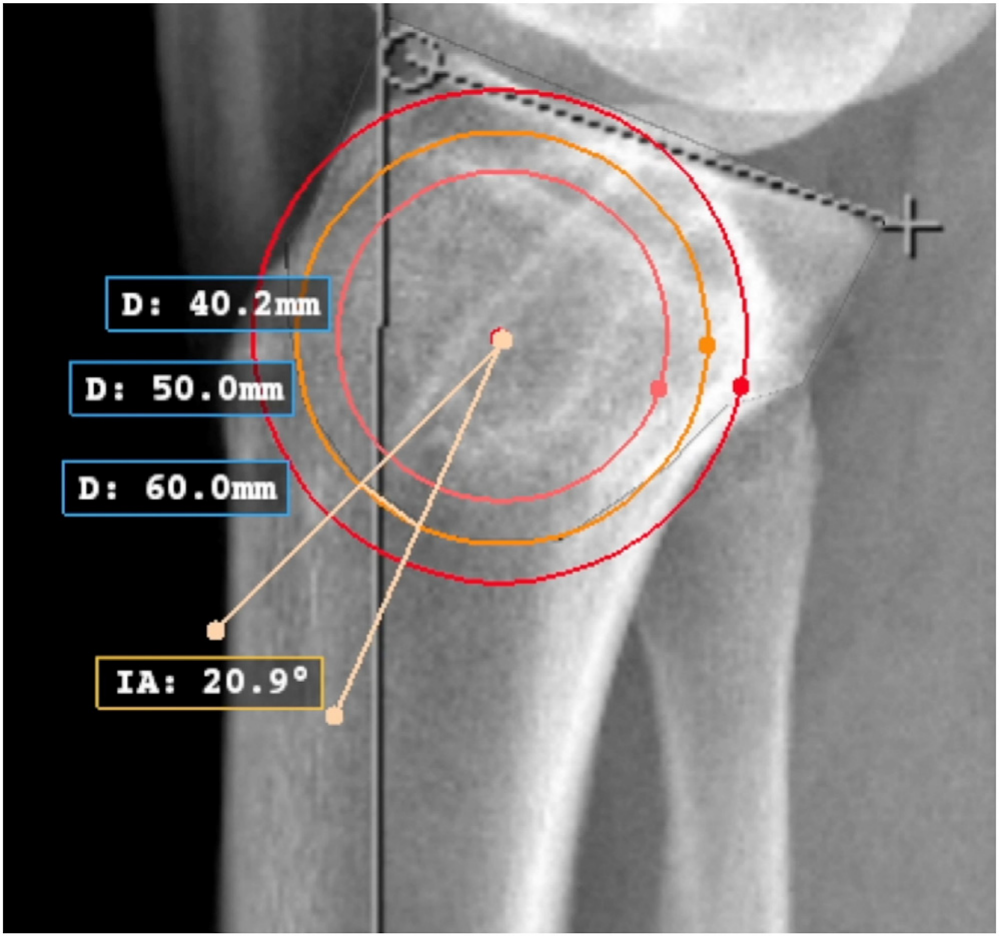
On a lateral radiograph of the right knee, 3 circles centered on the proximal tibial metaphysis are drawn, which represent possible osteotomy curves (red, orange, and pink circles). The radius of each circle should differ by 5 mm to identify what the optimal osteotomy size will be on the basis of unique patient characteristics (D: 40, 50, and 60 mm respectively). The optimal osteotomy curve starts just posterior to the tibial tubercle and ends just anterior to the posterior tibial cortex (orange circle). (D, diameter; IA, posterior tibial slope measure.)

**Fig 4:**
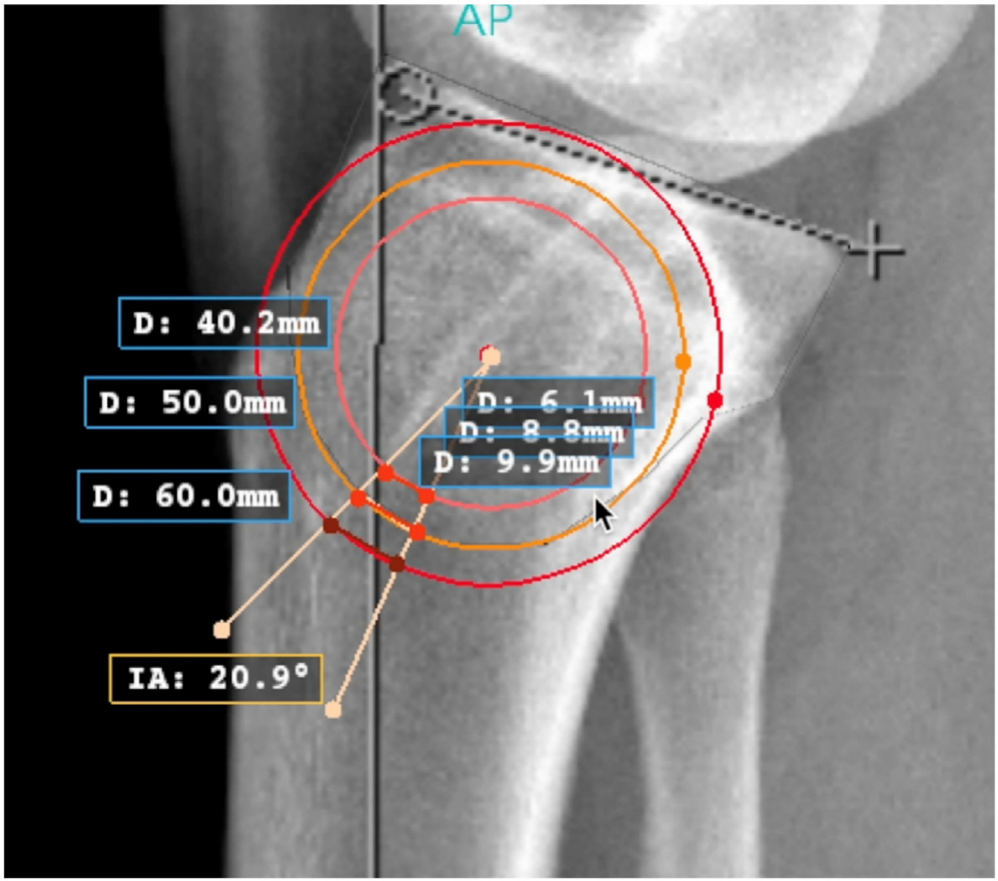
On a lateral radiograph of the right knee the desired correction (IA: 20.9°) is labeled. To measure the amount of rotational correction needed, measure the distance between the points at which each angle arm subtends a circle that represents a proposed osteotomy curve. If the red circle (D: 60 mm) is the template used to create the osteotomy, a rotational correction of (D: 9.9 mm) is needed to correct the slope (IA: 20.9°). Similarly, if the orange circle (D: 50 mm) represents the osteotomy, a rotational correction of (D: 8.8 mm) is needed. If the pink circle (D: 40 mm) represents the osteotomy, a rotational correction of (D: 6.1 mm) is needed. (D, diameter; IA, posterior tibial slope measure.)

Conventional knee or full-length tibia lateral radiographs and either the mechanical or anatomic axis may be used to measure the sagittal slope if a consistent method is used. The surgeon must be aware that using full-length tibia radiographs or the mechanical axis may result in slightly elevated slope measurements.^12^^,^^13^ On a perfect lateral radiograph, the center of the proximal tibia is identified by a best-fit circle (Fig 1). The osteotomy center of rotation is identified just above the proximal tibial-fibular joint (Fig 2). The angle of correction is defined by subtracting the desired postoperative slope from the preoperative slope. Three circles centered on the proximal tibial metaphysis are drawn, which represent possible osteotomy curves. The radius of each circle should differ by 5 mm to identify what the optimal osteotomy size will be on the basis of unique patient characteristics (Fig 3). The ideal osteotomy starts just posterior to the tibial tubercle and ends at the posterior cortex. The angle of correction is centered on the tibial metaphysis. The distance between the points at which each angle arm subtends a circle defines the amount of rotational correction needed to achieve the desired postoperative slope if that circle represents the osteotomy (Fig 4).

### Step 3: Surgical Positioning

After a general anesthetic is administered to the patient, an examination under anesthesia is completed. The patient is positioned supine with a nonsterile tourniquet placed proximally on the thigh. A lateral post and foot roll are placed to maintain a position of 90° of knee flexion and neutral hip rotation.

### Step 4: Diagnostic Arthroscopy

Diagnostic arthroscopy is performed. Preoperative imaging is correlated with intraoperative findings, specifically ACL and meniscus status. The amount of anterior tibial translation associated with large tibial slope may make any intra-articular work difficult before completion of the osteotomy.

### Step 5: Approach

**Fig 5:**
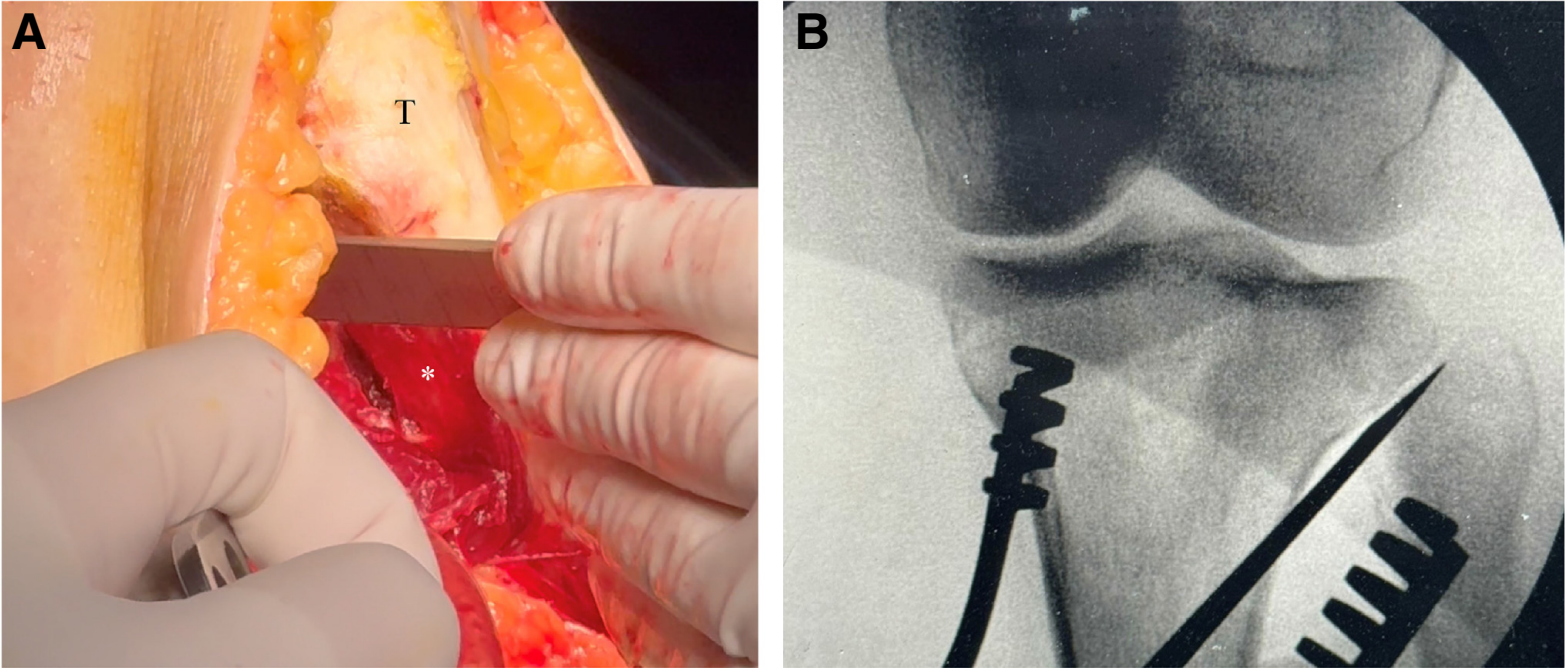
View of the anterolateral dissection of the right knee through a midline incision while the patient is in supine position with the knee flexed to 90°. (A) A midline incision is made over the tibial tubercle (T). Subperiosteal dissection of the anterior compartment (∗) provides access to the proximal tibial-fibular joint. (B) The joint capsule is incised and an osteotome is used to dissociate the joint, with care taken not to plunge posteriorly and injure the common peroneal nerve.

A midline longitudinal incision is centered over the tibial tubercle. Full-thickness fasciocutaneous flaps are elevated medially and laterally. Subperiosteal dissection of the anterior compartment is completed to the level of the proximal tibial-fibular joint. The proximal tibial-fibular joint capsule is incised and an osteotome is used to dissociate the joint, with care taken not to plunge posteriorly and injure the common peroneal nerve (Fig 5A and B). The dissection is continued medially just superficial to the medial collateral ligament to expose the complete medial aspect of the proximal tibia.

### Step 6: Osteotomy

**Fig 6:**
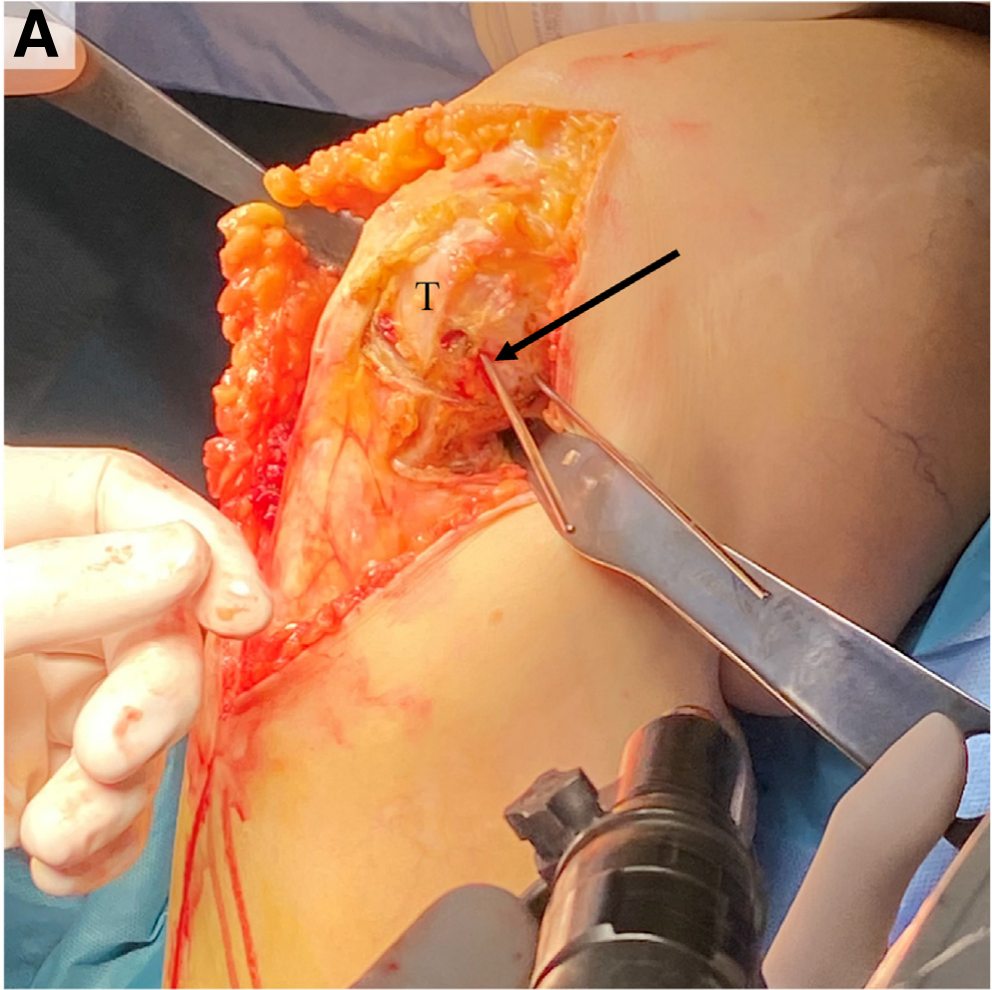
View from the medial aspect of the right knee through a midline incision with the patient supine and the knee flexed to 90° showing the placement of the central Kirshner wire (K-wire). Two protective Hohmann retractors are placed, one lateral and one posterior to the proximal tibia (T). A K-wire is placed at the center of the proximal tibia (black arrow).

**Fig 7:**
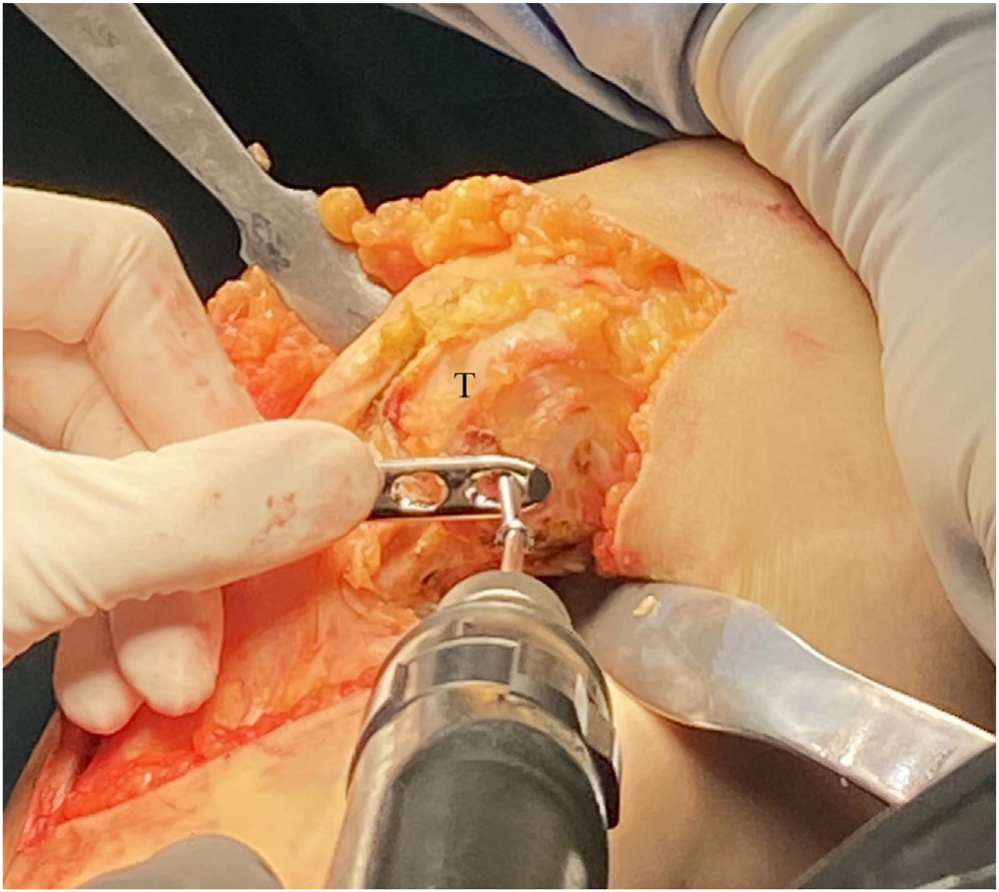
View from the medial aspect of the right knee through a midline incision with the patient supine and the knee flexed to 90°. The Kirshner wire is replaced by a 3.5-mm screw in the last hole of a standard straight 3.5mm locking plate after being drilled with a 2.5-mm drill in preparation for creation of a curved osteotomy in the proximal tibia (T).

**Fig 8:**
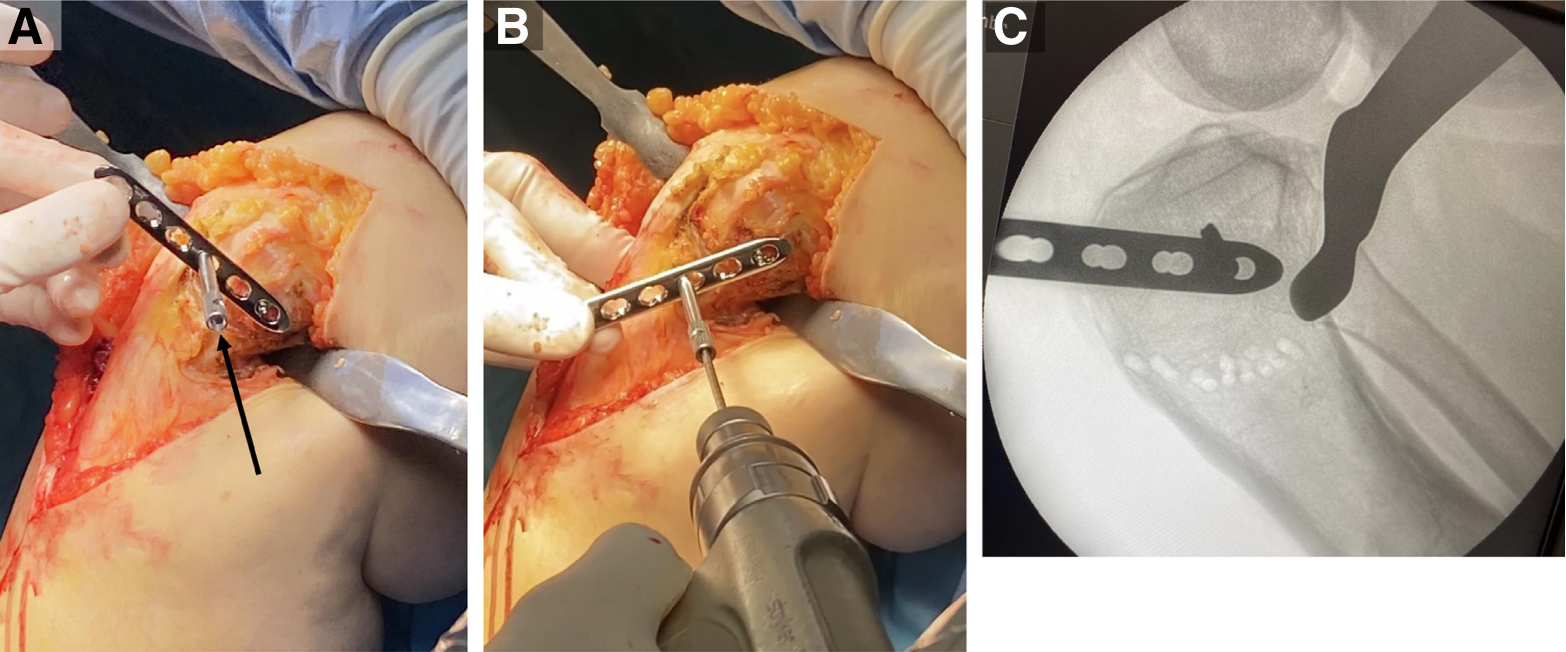
View from the medial aspect of the right knee through a midline incision with the patient supine and the knee flexed to 90° showing the preliminary osteotomy creation. (A) A locking guide is placed on the hole chosen to optimize the osteotomy curve based on preoperative planning (black arrow). (B) The proximal tibia is drilled from anterior just behind the tibial tubercle, to the posterior cortex in 2mm increments to outline a curved osteotomy. (C) A lateral fluoroscopic view of the right knee is used to confirm curved drill holes from anterior to posterior.

**Fig 9:**
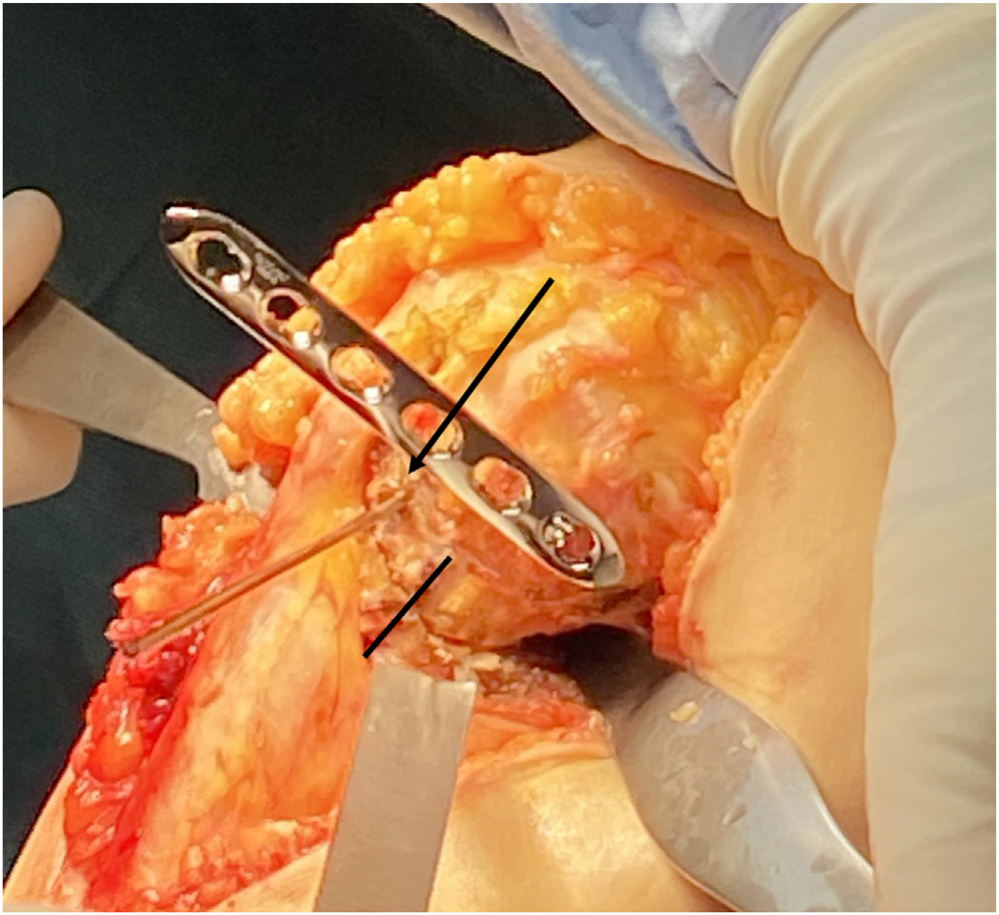
View from the medial aspect of the right knee through a midline incision with the patient supine and the knee flexed to 90°. The planned rotation distance to achieve the desired degree of correction is identified proximal-anterior with a K-wire (black arrow) and distal-posterior with a marking pen (black line).

**Fig 10:**
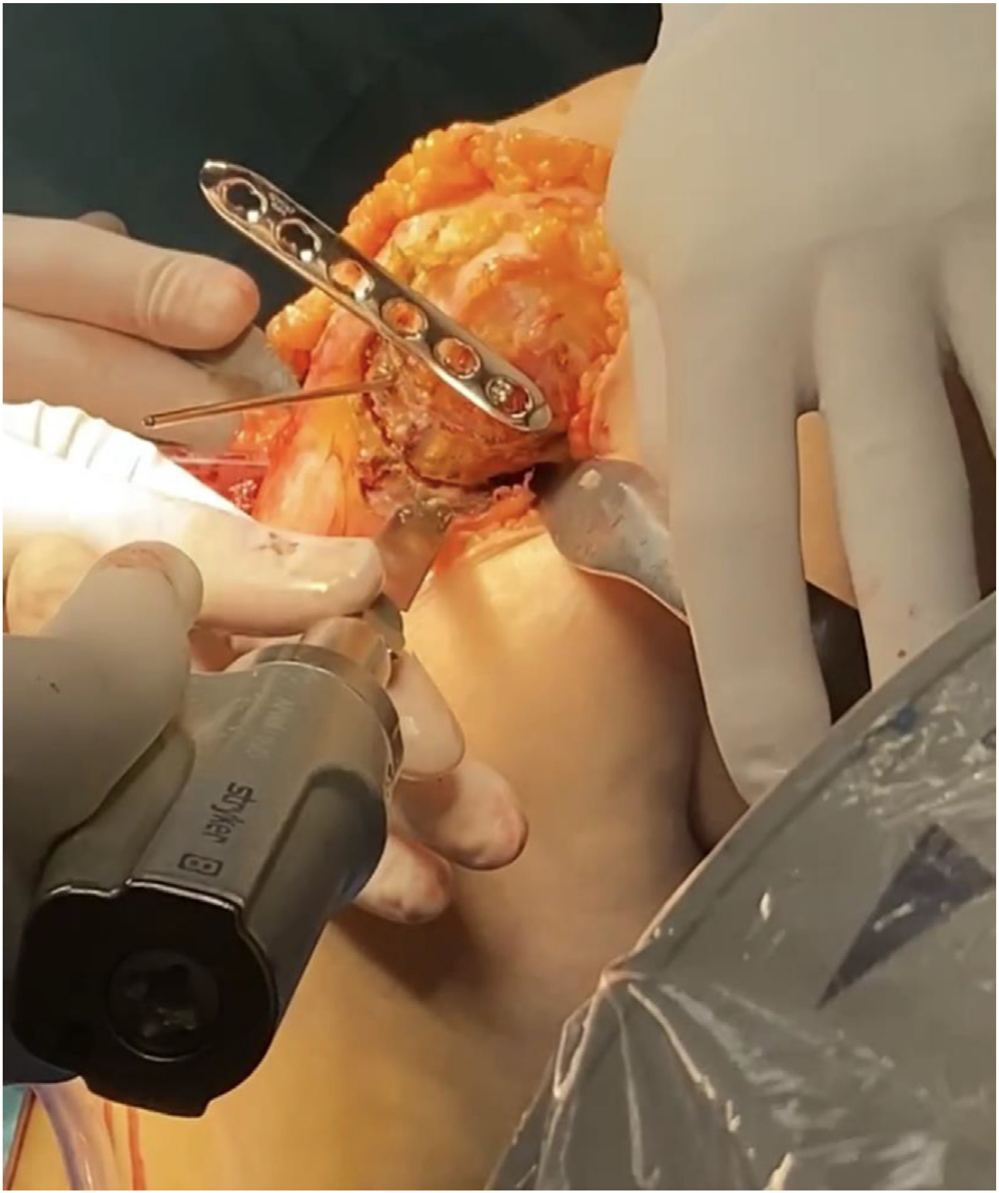
View from the medial aspect of the right knee through a midline incision with the patient supine and the knee flexed to 90°. The osteotomy is completed with a combination of the reciprocating saw and osteotomes while a Hohmann retractor protects the neurovascular bundle posteriorly.

Two protective Hohmann retractors are placed, one lateral and one posterior to the tibia from the medial side. A Kirshner wire (K-wire) is placed at the center of the proximal tibia, aided fluoroscopically with a perfect lateral radiograph (Fig 6). A 2.5-mm drill followed by 3.5-mm screw in the last hole of a standard straight 3.5-mm locking plate (DePuy Synthes, Raynham, MA) is placed at the center of the proximal tibia. The plate then functions as a drill guide to create a curved osteotomy (Fig 7). Each hole provides a different osteotomy radius, which should be optimized on the basis of preoperative planning. Electrocautery may be used to outline the osteotomy and cut the medial collateral ligament at the proposed osteotomy location. A locking guide is placed on the hole chosen to optimize the osteotomy curve and the proximal tibia is drilled from anterior to posterior in 2-mm increments to outline a curved osteotomy (Fig 8 A-C). The predetermined distance of rotation necessary to achieve desired degree of correction is marked proximal-anterior with a K-wire and distal-posterior with a marking pen (Fig 9). The osteotomy is completed with a combination of the reciprocating saw and osteotomes (Fig 10).

### Step 7: Reduction and Plate Osteosynthesis

**Fig 11:**
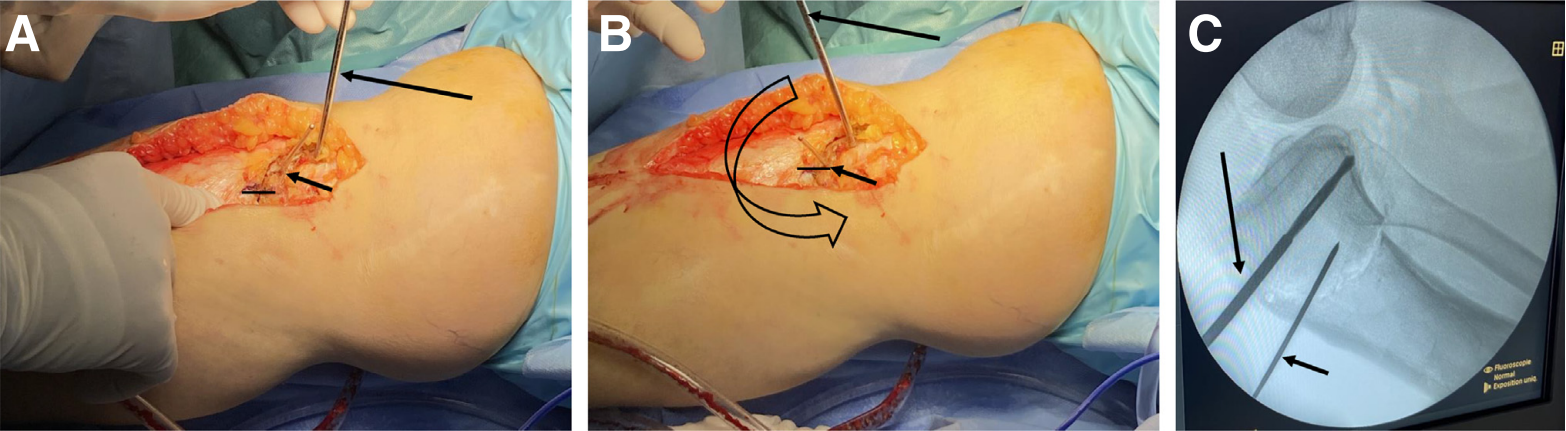
View from the medial aspect of the right knee through a midline incision with the patient supine and the knee in extension. Sagittal slope correction. (A) The slope correction is achieved by placing a 4-mm Steinman pin adjacent to the patellar tendon, anterior to posterior in the proximal segment once the osteotomy is mobile. (B) The distal segment is rotated anteriorly on the proximal segment using the Steinman pin to control the proximal segment position. (C) A lateral fluoroscopic assessment of the right knee shows the Steinman pin position and slope correction.

**Fig 12:**
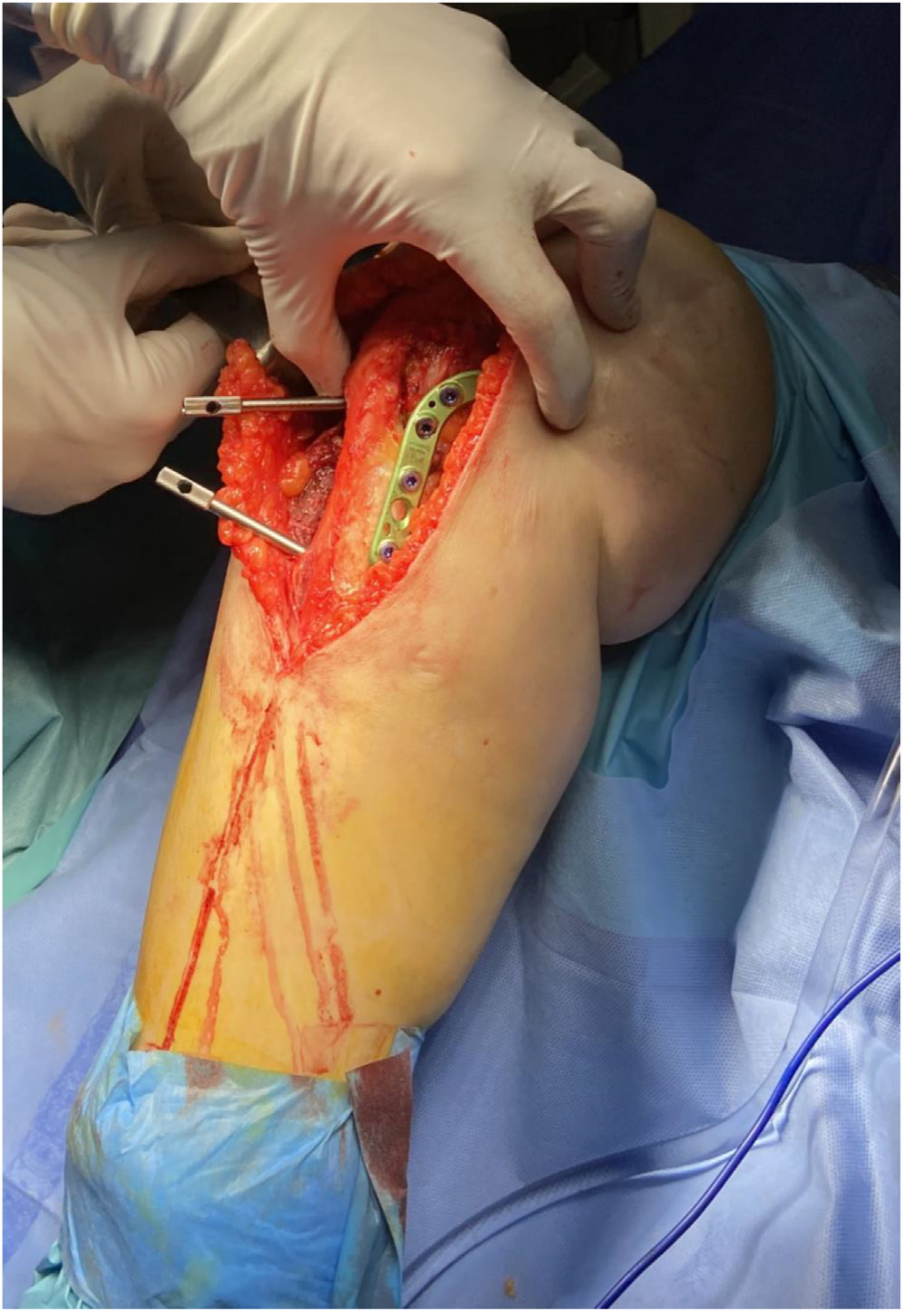
View from the anteromedial aspect of the right knee through a midline incision with the patient supine and the knee flexed to 90°. Dual plate and screw fixation are used on the anteromedial and anterolateral aspects to stabilize the osteotomy and allow for early range of motion and partial weight bearing.

**Fig 13:**
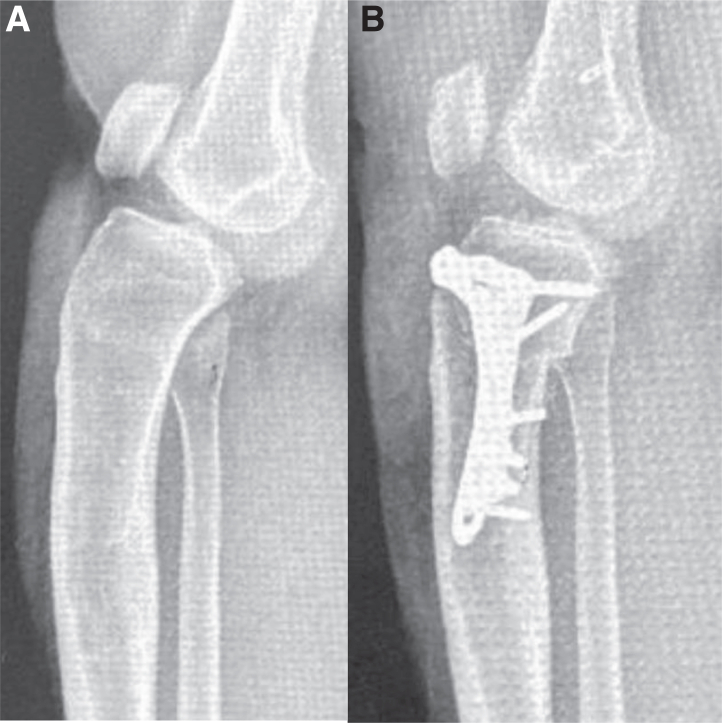
Lateral radiographs of the right knee. (A) A preoperative lateral radiograph of the right knee shows excessive proximal tibial sagittal slope with associated severe proximal tibial anterior subluxation and patella baja. (B) A 3-month postoperative lateral radiograph of the right knee shows large improvement of the sagittal proximal tibial slope, less anterior subluxation of the proximal tibia, and improved patellar height.

A 4-mm Steinman pin is placed adjacent to the patellar tendon, anterior to posterior in the proximal segment once the osteotomy is mobile. The distal segment is rotated anteriorly on the proximal segment using the Steinman pin to control the proximal segment position (Fig 11 A-C). Once the proximal-anterior K-wire is aligned with the distal-posterior pen mark, the desired rotational correction is achieved. Dual-plate and screw fixation are placed on the anteromedial and anterolateral aspects to stabilize the osteotomy and allow for early range of motion and partial weight bearing (Figs 12 and 13). Concomitant ACL and meniscus surgery may be performed simultaneously or in a staged fashion.

### Step 8: Postoperative Rehabilitation

The patient is placed in a posterior cruciate ligament-stabilizing brace to prevent recurvatum. The patient is permitted range of motion as tolerated and partial weight bearing for the first 6 weeks. Physical therapy commences on postoperative day 3. The patient may weight bear as tolerated at 6 weeks, if repeat radiographs show interval healing and maintained position of the osteotomy.

## Discussion

The optimal technique to decrease the sagittal slope of the proximal tibia is unknown. Although an ACWO is the commonly accepted technique, it may not be sufficient to correct a massively abnormal tibial slope. Advantages and disadvantages of different osteotomy strategies to reduce the proximal tibial sagittal slope are listed in Table 2. When the necessary correction is 20° or more, a TPLO may be preferred. A TPLO can achieve a large correction at the level of the metaphysis while still respecting the tibial tubercle without altering patellar height. The metaphyseal location improves healing compared with a diaphyseal osteotomy.^14^ Furthermore, a curved osteotomy has the advantage of increased surface area for bony contact, which also facilitates bony healing.^15^ For these reasons, the TPLO remains the osteotomy of choice to treat cranial-cruciate ligament rupture in canines.^9^ Perhaps in the scenario of a massively increased slope, such as after asymmetric posterior proximal tibial physeal arrest, a TPLO should be the osteotomy of choice to achieve satisfactory slope correction.

**Table 2:** Advantages and Disadvantages of Different Osteotomy Strategies to Reduce the Proximal Tibial Sagittal Slope

	Advantages	Disadvantages
Supratubercular ACWO	- Metaphyseal location has high healing potential - Coronal plane correction with an asymmetric osteotomy is possible - Smaller incision, less invasive	- Technically demanding - Limitation to the amount of slope correction possible - Risk of intra-articular extension - Large corrections may result in patella alta
Transtubercular ACWO	- Technically easier - Metaphyseal location has high healing potential - Coronal plane correction with an asymmetric osteotomy is possible	- Added morbidity of associated tibial tubercle osteotomy - Slower recovery because of prolonged mandatory extension postoperatively - Large corrections may result in proximal tibia recurvatum - Large corrections may result in patella alta
Infratubercular ACWO	- Potential for large correction	- Increased risk for nonunion resulting from the diaphyseal osteotomy site - Necessary large anterior tibial diaphyseal resection carries a high risk of secondary tibial plateau fracture - Difficult to complete an asymmetric osteotomy in the coronal plane - Large corrections may result in tibial recurvatum -
Tibial plateau-leveling osteotomy	- Large corrections are possible - No change in patella height - No tibial angular deformity is introduced - Improved soft-tissue balancing compared with ACWO - Metaphyseal location has high healing potential	- Technically demanding and learning curve present - Exceedingly difficult to complete an asymmetric osteotomy in the coronal plane

ACWO, anterior closing-wedge osteotomy.

This report describes the rationale, technique, and limitations of a TPLO. Benefits include the potential for a large rotational correction with no change in patella height or introduction of tibial angular deformity. Limitations include the initial learning curve and unfamiliar surgery and difficulty completing a simultaneous asymmetric coronal plane correction if needed.

## Disclosures

The authors declare the following financial interests/personal relationships which may be considered as potential competing interests: M.O. reports consulting or advisory with Newclip Technics. K.K. reports consulting or advisory with Newclip Technics. J.B. reports consulting or advisory with Newclip Technics. All other authors (A.J.H., S.O., S.F-O., B.G.) declare that they have no known competing financial interests or personal relationships that could have appeared to influence the work reported in this paper.

## Supplementary Data

Video 1 Tibial plateau-leveling osteotomy of the right knee in supine position. Preoperative planning considers the preoperative sagittal slope, desired correction, and optimal osteotomy radius of curvature. A midline longitudinal incision is made with full-thickness fasciocutaneous flaps. The tibialis anterior is elevated subperiosteally and the proximal tibial-fibular joint is dissociated with an osteotome. The medial dissection is just superficial to the medial collateral ligament to expose the complete medial aspect of the proximal tibia. The center of the proximal tibia is identified on a true lateral radiograph with a Kirshner wire and replaced with a 3.5-mm screw through the last hole of a standard 3.5-mm locking plate. A locking guide is placed on the plate hole that corresponds to the preplanned radius of curvature of the osteotomy and a curved osteotomy is drilled in 2-mm increments through the locking guide. The preplanned rotational correction is marked. The osteotomy is completed with a combination reciprocating saw and osteotomes. A 4-mm Steinman pin is placed in the proximal segment adjacent to the patellar tendon and the proximal segment is stabilized while the distal segment is rotated anteriorly in extension to match the planned correction. The osteotomy is stabilized with dual anteromedial and anterolateral plates to allow for early mobilization and weight bearing.
